# Cross-Protection against MERS-CoV by Prime-Boost Vaccination Using Viral Spike DNA and Protein

**DOI:** 10.1128/JVI.01176-20

**Published:** 2020-11-23

**Authors:** Jung-ah Choi, Junghyun Goo, Eunji Yang, Dae-Im Jung, Sena Lee, Semi Rho, Yuji Jeong, Young-Shin Park, Hayan Park, Young-hye Moon, Uni Park, Sang-Hwan Seo, Hyeja Lee, Jae Myun Lee, Nam-Hyuk Cho, Manki Song, Jae-Ouk Kim

**Affiliations:** a Science Unit, International Vaccine Institute, Seoul, South Korea; b Department of Microbiology and Immunology, Seoul National University College of Medicine, Seoul, South Korea; c Department of Biomedical Sciences, Seoul National University College of Medicine, Seoul, South Korea; d NKMAX Co., Ltd., Sungnam, South Korea; e Department of Microbiology and Immunology, Graduate School of Medical Science, Brain Korea 21 Project, Institute for Immunology and Immunological Diseases, Yonsei University College of Medicine, Seoul, South Korea; Loyola University Chicago

**Keywords:** MERS-CoV, vaccines

## Abstract

Middle East respiratory syndrome coronavirus (MERS-CoV) causes severe respiratory illness and has a high mortality of ∼34%. However, since its discovery in 2012, an effective vaccine has not been developed for it. To develop a vaccine against multiple strains of MERS-CoV, we targeted spike glycoprotein (S) using prime-boost vaccination with DNA and insect cell-expressed recombinant proteins for the receptor-binding domain (RBD), S1, S2, SΔTM, or SΔER. Our S subunits were generated using an S sequence derived from the MERS-CoV EMC/2012 strain. We examined humoral and cellular immune responses of various combinations with DNA plasmids and recombinant proteins in mice. Mouse sera immunized with SΔER DNA priming/SΔTM protein boosting showed cross-neutralization against 15 variants of S-pseudovirions and the wild-type KOR/KNIH/002 strain. In addition, these immunizations provided full protection against the KOR/KNIH/002 strain challenge in human DPP4 knock-in mice. These findings suggest that vaccination with the S subunits derived from one viral strain can provide cross-protection against variant MERS-CoV strains with mutations in S. DNA priming/protein boosting increased gamma interferon production, while protein-alone immunization did not. The RBD subunit alone was insufficient to induce neutralizing antibodies, suggesting the importance of structural conformation. In conclusion, heterologous DNA priming with protein boosting is an effective way to induce both neutralizing antibodies and cell-mediated immune responses for MERS-CoV vaccine development. This study suggests a strategy for selecting a suitable platform for developing vaccines against MERS-CoV or other emerging coronaviruses.

**IMPORTANCE** Coronavirus is an RNA virus with a higher mutation rate than DNA viruses. Therefore, a mutation in S-protein, which mediates viral infection by binding to a human cellular receptor, is expected to cause difficulties in vaccine development. Given that DNA-protein vaccines promote stronger cell-mediated immune responses than protein-only vaccination, we immunized mice with various combinations of DNA priming and protein boosting using the S-subunit sequences of the MERS-CoV EMC/2012 strain. We demonstrated a cross-protective effect against wild-type KOR/KNIH/002, a strain with two mutations in the S amino acids, including one in its RBD. The vaccine also provided cross-neutralization against 15 different S-pseudotyped viruses. These suggested that a vaccine targeting one variant of S can provide cross-protection against multiple viral strains with mutations in S. The regimen of DNA priming/Protein boosting can be applied to the development of other coronavirus vaccines.

## Abstract (precis)

Coronavirus is an RNA virus with a higher mutation rate than DNA viruses. Therefore, a mutation in S-protein, which mediates viral infection by binding to a human cellular receptor, is expected to cause difficulties in vaccine development. Given that DNA-protein vaccines promote stronger cell-mediated immune responses than protein-only vaccination, we immunized mice with various combinations of DNA priming and protein boosting using the S-subunit sequences of the MERS-CoV EMC/2012 strain. We demonstrated a cross-protective effect against wild-type KOR/KNIH/002, a strain with two mutations in the S amino acids, including one in its RBD. The vaccine also provided cross-neutralization against 15 different S-pseudotyped viruses. These suggested that a vaccine targeting one variant of S can provide cross-protection against multiple viral strains with mutations in S. The regimen of DNA priming/Protein boosting can be applied to the development of other coronavirus vaccines.

## INTRODUCTION

Middle East respiratory syndrome coronavirus (MERS-CoV) was first identified in the Kingdom of Saudi Arabia in 2012 and has been causing recurrent infectious outbreaks of respiratory illness in humans (1). At the end of January 2020, 2,519 laboratory-confirmed cases of MERS worldwide, including 866 associated deaths and a mortality rate of 34.3%, were reported. MERS is a zoonotic disease with bats and dromedary camels, playing an important role in its emergence (2). MERS-CoV is transmitted to humans through close contact with dromedaries (3). Vaccination is expected to be an efficacious strategy in preventing individuals and animals against contracting MERS-CoV infections, but no vaccine or specific treatment for MERS has been globally approved yet. To date, several MERS-CoV vaccine candidates have been developed, including DNA, subunit protein, nanoparticle, inactivated whole-virus, and recombinant viral vector-based such as adenoviral vectors, modified vaccinia virus Ankara, and recombinant measles virus (4–6).

MERS-CoV is an enveloped virus with a positive-sense single-stranded RNA genome (7). Among the four structural proteins of MERS-CoV spike (S), envelope, membrane, and nucleocapsid, the S glycoprotein is expected as the candidate molecule for an appropriate vaccine to induce neutralizing antibodies (8). S is a class 1 viral fusion protein that mediates host receptor attachment and fusion of the viral and cellular membranes. S is trimeric, and each protomer is synthesized as a single polypeptide chain of 1,395 amino acids. The S glycoprotein is cleaved into the receptor-binding subunit S1 and the membrane fusion subunit S2 by host proteases during the infection process (9–11). S1 and S2 remain noncovalently bound in the prefusion conformation (12). The S1 subunit comprises the apex of the S trimer, including the receptor-binding domains (RBDs), and stabilizes the prefusion state of the S2 fusion machinery, which is anchored in the viral membrane. S is further cleaved by host proteases at the so-called “S2” site located immediately upstream of the fusion peptide. This cleavage has been proposed to activate the protein for membrane fusion via large-scale, irreversible conformational changes.

Most recombinant vaccine candidates use full-length S or its truncated version of S1. Because the conformation of RBD in full-length S and truncated versions may differ, the recombinant RBD subunit protein itself may not induce neutralizing antibodies as efficiently as a larger subunit such as S1 or transmembrane deleted S (SΔTM) (13). Hence, the goal of our study was to systematically investigate the effect of S subunits, including RBD, S1, S2, and SΔTM, and the heterologous prime-boost regimen on protective immune responses in mice.

In this study, we produced DNA plasmids encoding several S subunits and recombinant proteins in insect cells for RBD, S1, S2, and SΔTM and compared the protective immune responses of various combinations in mice. The results of this study are expected to contribute to the development of an appropriate MERS-CoV vaccine strategy using DNA prime-protein boost with the S subunit.

## RESULTS

### Selection of MERS-CoV SΔER as a DNA vaccine vector. (i) Construction of MERS-CoV S subunit DNA plasmids.

To examine the immunogenicity of MERS-CoV S subunits as a DNA vaccine, human codon-optimized pSΔER, pSΔTM, pS1, pRBD, and pS2 DNA were generated using a mammalian expression vector. pSΔER (amino acids [aa] 1 to 1338) has a truncated S gene, including the deletion of the endoplasmic reticulum (ER) retention signal (aa 1339 to 1353), pSΔTM (aa 1 to 1296) with a deletion from the transmembrane domain, pS1 (aa 1 to 751), pRBD (aa 1 to 18 and 358 to 606), and pS2 (aa 1 to 18 and 752 to 1296). Both pRBD and pS2 have an additional signal peptide (aa 1 to 18) sequence at the N terminus to facilitate the extracellular secretion of recombinant proteins.

**FIG 1:**
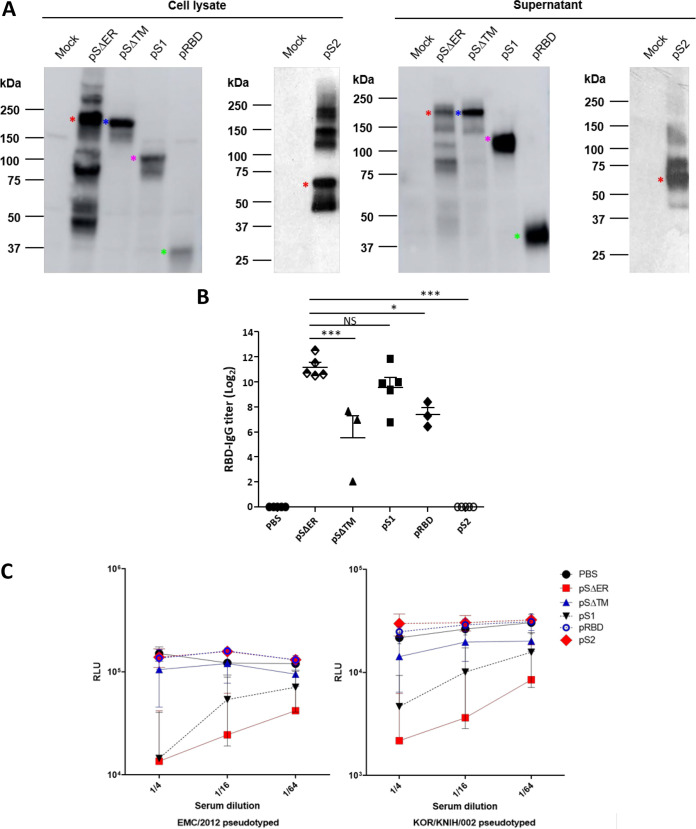
Comparison of immunogenicity induced by various MERS-CoV S subunit DNA constructs. (A) S subunit protein expression in 293T cells, includingsecretion into cell culture supernatants, was confirmed by Western blotting after transfection with each DNA construct. (Mock, Mock plasmid; pSΔER, SΔER plasmid; pSΔTM, SΔTM plasmid; pS1, S1 plasmid; pRBD, RBD (+signal peptide) plasmid; pS2, S2 (ΔTM + signal peptide) plasmid. (B and C) Immunogenicity of various MERS-CoV S subunit DNA constructs. BALB/c mice (*n *=* *5) were i.m. immunized with 50 μg of pSΔER, pSΔTM, pS1, pRBD, and pS2 DNA or 50 μl of PBS three times (at days 0, 14, and 28). Sera were collected 14 days after the third immunization (at day 42), and RBD protein-specific serum IgG level was measured by ELISA. Significant differences are indicated as follows: *, *P* < 0.05; **, *P* < 0.01; ***, *P* < 0.001; and NS, not significant. (B). Neutralizing activity of 1/4-, 1/16-, and 1/64-diluted sera against MERS-CoV EMC/2012 and KOR/KNIH/002 S-pseudovirions was analyzed by measuring luciferase activity (C). The results are expressed as means ± the standard deviations (SD). Significant differences are indicated as follows: *, *P* < 0.05; **, *P* < 0.01; ***, *P* < 0.001; and ****, *P* < 0.0001.

S-protein expression from pSΔER, pSΔTM, pS1, pRBD, or pS2 DNA was verified by Western blot analysis. HEK293T cells were transfected with each DNA plasmid or mock vector as the negative control. The bands corresponding to the MERS-CoV SΔER protein (∼148 kDa), SΔTM protein (∼143 kDa), S1 protein (∼94 kDa), RBD protein (∼27 kDa), and S2 protein (∼66 kDa) in cell lysates and cell culture supernatants were detected (Fig. 1A). In cell lysates, pSΔER transfection exhibited a higher expression level of corresponding protein than pSΔTM, pS1, and pRBD transfection. The bands of the SΔER protein with a size larger than expected were assumed glycosylated proteins and those with a smaller size as cleaved proteins. In contrast to the protein expression level in cell lysates, pS1 and pRBD transfection showed even higher expression levels of secreted proteins in cell culture supernatant than pSΔER and pSΔTM. Interestingly, pSΔER showed the band with a size similar to that of pSΔTM and other smaller bands in cell culture supernatant, indicating the secretion of proteins. pS2 showed the bands with a size larger and smaller than expected as well as in the supernatant. These results indicated that recombinant SΔTM, S1, RBD, and S2 proteins were expressed by the transfected cells and secreted.

### (ii) Antibody responses induced by MERS-CoV S subunit DNA plasmids.

To investigate the level of *in vivo* humoral immune responses induced by various MERS-CoV S subunit DNA vaccines, 50 μg of each DNA vector was administered via the intramuscular (i.m.) route three times at 2-week intervals. Sera were collected 2 weeks after the last immunization and assessed for the presence of MERS-CoV RBD-specific antibody by ELISA. As expected, RBD protein (358 to 606 aa)-specific antibody responses were not detected in the mice immunized with pS2 (1 to 18 and 752 to 1,296 aa) but were present in those immunized with pSΔER, pSΔTM, pS1, and pRBD DNA (Fig. 1B). The pSΔER DNA-immunized group presented a higher anti-RBD IgG titer than pSΔTM and pRBD DNA-immunized group. There was no statistically significant difference between the pSΔER and pS1 group, but the mean value of the pSΔER group was higher than that of the pS1 group.

Neutralizing activity was determined using the EMC/2012 and KOR/KNIH/002 strains of MERS-CoV pseudovirion containing the luciferase reporter gene. Diluted sera were incubated with each pseudovirion, and inhibition of pseudovirus entry into target cells was assessed by measuring luciferase activity in cell lysates. The results were expressed as relative luciferase units (RLU). Lower RLU value indicated a higher level of inhibition of pseudovirion infection into the cells. pSΔER and pS1 DNA immunization induced a statistically significant increase in neutralizing antibody in the sera (*P* < 0.05 at all of the serum dilutions), but the pSΔTM, pRBD, and pS2 DNA-immunized mice did not show statistically significant differences compared to phosphate-buffered saline (PBS)-administered mice (Fig. 1C). In both EMC/2012 and KOR/KNIH/002 strains, pSΔER DNA-immunized mice showed the highest neutralizing activity compared to the other DNA-immunized groups. These results indicate that the SΔER DNA plasmid is the most effective construct to induce antibody immune responses in mice. Therefore, pSΔER was selected as the final DNA vaccine vector to be used for DNA priming.

### pSΔER DNA prime-SΔTM protein boost induced comparable humoral immune responses to S1 and SΔTM protein subunits.

**FIG 2:**
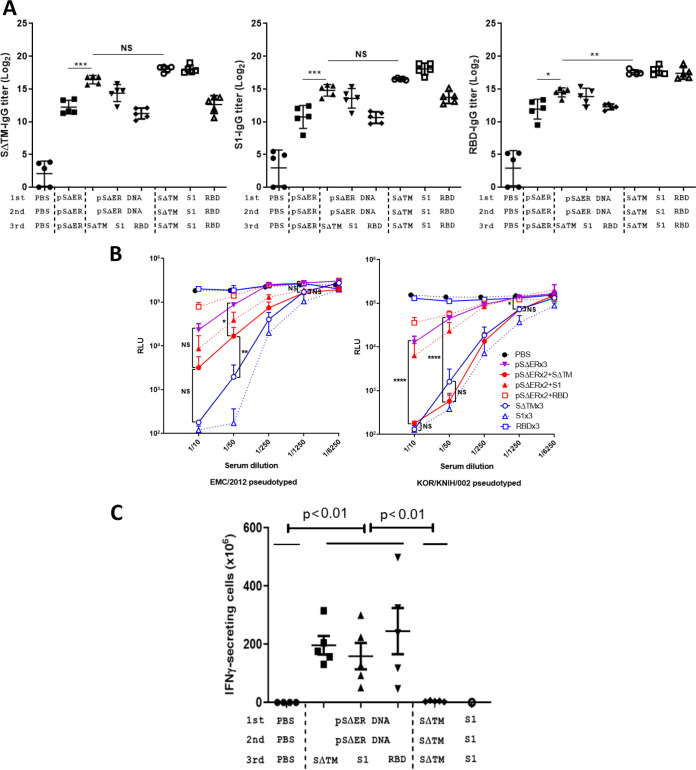
Humoral and cellular immune responses induced by immunization with pSΔER DNA/various recombinant MERS-CoV S subunit proteins. Enhancedimmunogenicity by priming with pSΔER DNA and boosting with various recombinant MERS-CoV S subunit proteins. (A) Antibody responses induced by DNA prime-protein boost. BALB/c mice (*n *=* *5) were i.m. immunized with pSΔER DNA (50 μg) two times, followed by various S-proteins (SΔTM, S1, S2, and RBD, 1 μg each), and pSΔER DNA (50 μg), SΔTM protein (1 μg), S1 protein (1 μg), RBD protein (1 μg), or PBS three times as controls. All of the proteins were adjuvanted with alum hydroxide. Mouse sera were collected 14 days after the last immunization (at day 42), and their titers were analyzed by ELISA. Serum IgG titers were measured against SΔTM, S1, and RBD proteins. IgG titer is expressed as the reciprocal log_2_ of serum dilution showing an absorbance of 0.2 at 450 nm. The results are expressed as means ± the SD. Significant differences are indicated as follows: *, *P* < 0.05; **, *P* < 0.01; ***, *P* < 0.001; and NS, not significant. Statistical significance was compared between the pSΔER DNA-immunized group, or SΔTM protein-immunized group against the pSΔER DNA prime-SΔTM protein boost immunized group. (B) Neutralizing activity of DNA prime-protein boost vaccine against with MERS-CoV vaccine. The neutralizing effect of serially diluted sera (1/10, 1/50, 1/250, 1/1,250, and 1/6,250) against EMC/2012 S-pseudovirions and KOR/KNIH/002 S-pseudovirions was assessed by measuring luciferase activity. The results are indicated as means ± the SD. Significant differences are indicated as follows: *, *P* < 0.05; **, *P* < 0.01; ***, *P* < 0.001; NS, not significant. (C) T-cell immune response induced by DNA prime–protein boost with MERS-CoV vaccine. Vaccinated C57BL/6 mice were sacrificed 14 days after the last immunization (at day 42), and splenocytes were stimulated with S1, RBD, and S2 pooled peptides. IFN-γ-producing T cells were enumerated by using an ELISpot assay. There were statistically significant differences between the PBS group versus all three DNA prime-protein boost groups and the SΔTM protein group (two-tail Mann-Whitney U test). The results are expressed as means ± the SD.

To examine the effect of boosting with various S subunit proteins after DNA priming, recombinant SΔTM (1 to 1,296 aa), S1 (1 to 751 aa), S2 (752 to 1,296 aa), and RBD (358 to 606 aa) proteins were produced in SF9 insect cells by using the baculovirus system with a protein purity of ≥85%, as described previously (14). Mice were immunized i.m. with DNA only, DNA prime followed by a protein boost, or protein only. The following combinations of MERS-CoV S DNA and/or proteins were used: (i) three times with pSΔER DNA; (ii) pSΔER DNA two times, followed by various S-subunit proteins (SΔTM, S1, S2, and RBD); (iii) SΔTM protein, S1 protein, and RBD protein; or (iv) PBS as a negative control. All the proteins were adjuvanted with alum hydroxide. Serum samples were collected 2 weeks after the last immunization, and SΔTM-, S1-, and RBD-specific antibody titers in mice sera were determined by ELISA. As shown in Fig. 2A, the mice immunized with pSΔER DNA prime (two doses)–SΔTM protein boost (one dose) showed the highest antibody titers against each of the SΔTM, S1, and RBD antigens within the DNA prime-protein boost-vaccinated group. SΔTM- and S1-specific antibody titers of pSΔER DNA prime-SΔTM protein boost-vaccinated group were significantly higher than those of the pSΔER DNA-vaccinated group but were not statistically significant compared to those of the SΔTM protein-vaccinated group. The RBD-specific antibody titer of the pSΔER DNA prime-SΔTM protein boost-vaccinated group was higher than that of the pSΔER DNA-vaccinated group and lower than that of the SΔTM protein-vaccinated group.

In addition, neutralizing activity was determined using S-pseudovirions obtained from MERS-CoV EMC/2012 and KOR/KNIH/002 strains. Similar to the results of the antibody titer, the mice immunized with pSΔER DNA prime-SΔTM protein boost vaccination showed the highest neutralizing activity among the DNA prime-protein boost groups (Fig. 2B). The neutralizing activity of pSΔER DNA prime–SΔTM protein boost-vaccinated group was higher than that of the pSΔER DNA-vaccinated group at a 1/50 serum dilution and comparable to that of the SΔTM protein-vaccinated group (1/10 dilution, no significance; 1:50 dilution, *P* < 0.01). However, the RBD protein-vaccinated group showed minimal neutralizing activity against the S-pseudovirions of both EMC/2012 and KOR/KNIH/002 strains, while RBD-binding IgG titer was not statistically different from that of SΔTM- and S1-protein vaccination. These data demonstrated that immunization with the pSΔER DNA prime-SΔTM protein is an effective vaccination regimen to induce potent humoral immune responses in mice. Similarly, vaccination with SΔTM or S1 protein also induced robust humoral responses.

### pSΔER DNA prime-SΔTM protein boost elevated cell-mediated immune response.

Cell-mediated immune responses were evaluated by determining the levels of cytokines secreted by cells. Gamma interferon (IFN-γ) is the typical Th1-type cytokine and is produced by CD4^+^ and CD8^+^ T cells. The IFN-γ level was measured by a mouse IFN-γ enzyme-linked immunosorbent spot (ELISpot) assay. MERS-CoV S pooled peptide was used for eliciting S-protein-specific CD8^+^ T-cell response. Unrelated gag peptide was used as the negative control, and PMA/ionomycin was used as a positive control. T-cell responses were measured 14 days after the last vaccination. The mice vaccinated with DNA prime-protein boost vaccine showed S pooled peptide-specific CD8^+^ T-cell-mediated immune response as determined by IFN-γ-secreting splenocyte counts (Fig. 2C). However, the protein subunit-vaccinated group showed no IFN-γ-secreting cells. These data indicated that the pSΔER DNA prime-SΔTM protein boost vaccine generated a higher T-cell-mediated immune response.

### SΔER DNA prime-SΔTM protein boost induced neutralizing antibodies against wild-type MERS-CoV strain.

**FIG 3:**
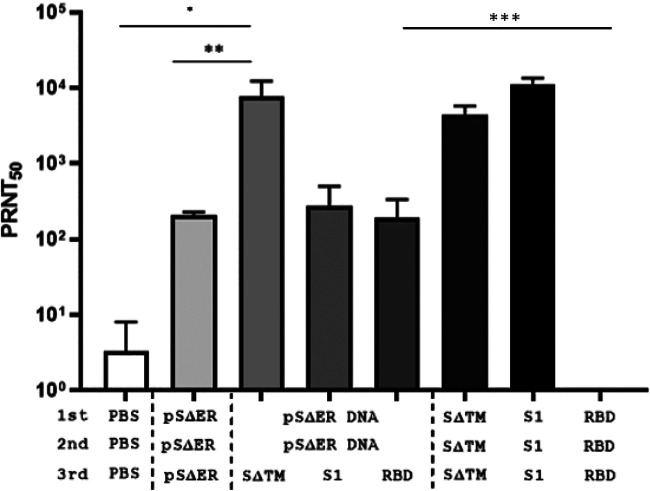
*In vitro* neutralizing effect of MERS-CoV vaccine against the wild-type MERS-CoV strain. Serially diluted mouse sera were incubated with the wild-type MERS-CoV KOR/KNIH/002 strain, and the mixture was added to VeroE6 cells. After 3 days, the plaque numbers were counted, and the last dilution factor showing a value greater than 50% is expressed as the PRNT_50_ titer. Statistical analyses of each group were performed with a two-tailed unpaired test. The results are expressed as means ± the SD. Significant differences are indicated as follows: *, *P* < 0.05; **, *P* < 0.01; and ***, *P* < 0.001. There was no statistical significance between the experimental groups except for the statistically marked experimental group.

To determine the protective immune response against natural infection, the neutralizing antibody titer against wild-type MERS-CoV KOR/KNIH/002 strain was determined based on the 50% plaque reduction/neutralization titer (PRNT_50_). The results showed a pattern similar to the neutralizing activity against pseudovirions described earlier. The mice immunized with the pSΔER DNA prime–SΔTM protein boost showed the highest wild-type virus-neutralizing activity among the DNA prime–protein boost-vaccinated groups (Fig. 3). The wild-type virus-neutralizing activity in the pSΔER DNA prime-SΔTM protein boost group was higher than that in the pSΔER DNA-treated group and comparable to the SΔTM protein-treated or S1 protein-treated group. However, the RBD protein-vaccinated group did not show neutralizing activity against the wild-type MERS-CoV strain. Therefore, these results indicated that our pSΔER DNA prime-SΔTM protein boost vaccine induced neutralizing antibodies against the wild-type MERS-CoV strain comparable to adjuvanted recombinant SΔTM and S1 protein.

### pSΔER DNA prime-SΔTM protein boost induced cross-protective neutralizing activity against various MERS-CoV S-pseudovirions.

**FIG 4:**
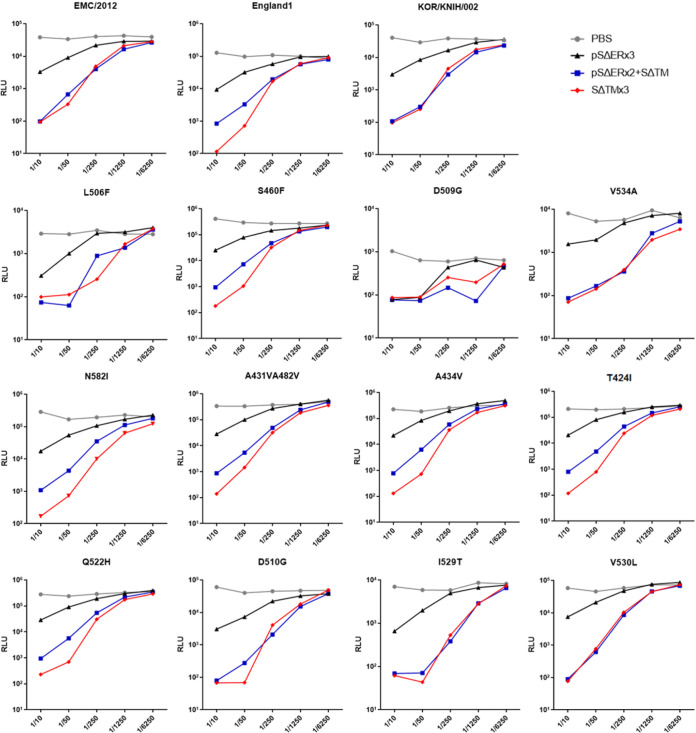
Evaluation of cross-neutralizing activities against various MERS-CoV S-pseudovirions. The neutralizing activities of the DNA prime-protein boost vaccine, protein vaccine, and DNA vaccine were assessed. Diluted mouse sera (1/10, 1/50, 1/250, 1/1,250, and 1/6,250) were tested for their neutralizing activities against 15 pseudovirions by measuring the luciferase activities of target cells.

To evaluate the cross-protective neutralizing activity of DNA prime-protein boost vaccination, we performed a pseudovirus neutralization assay against 15 different MERS-CoV S-pseudovirions. Fifteen MERS-CoV S gene sequences were selected from GenBank. Three pseudovirions were constructed using each DNA plasmid encoding SΔER of EMC/2012, KOR/KNIH/002, and England1, and 12 of them were constructed based on the EMC/2012 strain S sequence with mutations only in the RBD region as described previously (2). Sera from pSΔER DNA prime-SΔTM protein boost-, pSΔER DNA-, and SΔTM protein-immunized group were tested against each pseudovirion. The neutralizing activity of pSΔER DNA prime-SΔTM protein boost was comparable to that of SΔTM protein vaccination and higher than that of pSΔER DNA immunization against all the 15 pseudovirions. We demonstrated that pSΔER DNA prime-SΔTM protein boost vaccine could protect against infection caused by various MERS-CoV S-pseudovirions (Fig. 4). Thus, our pSΔER DNA prime-SΔTM protein boost vaccination is expected to be cross-protective against MERS-CoVs with mutations in the S, which mediates viral entry to the cells.

### SΔER DNA prime-SΔTM protein boost vaccine exhibited a protective effect against wild-type MERS-CoV infection in mice.

**FIG 5:**
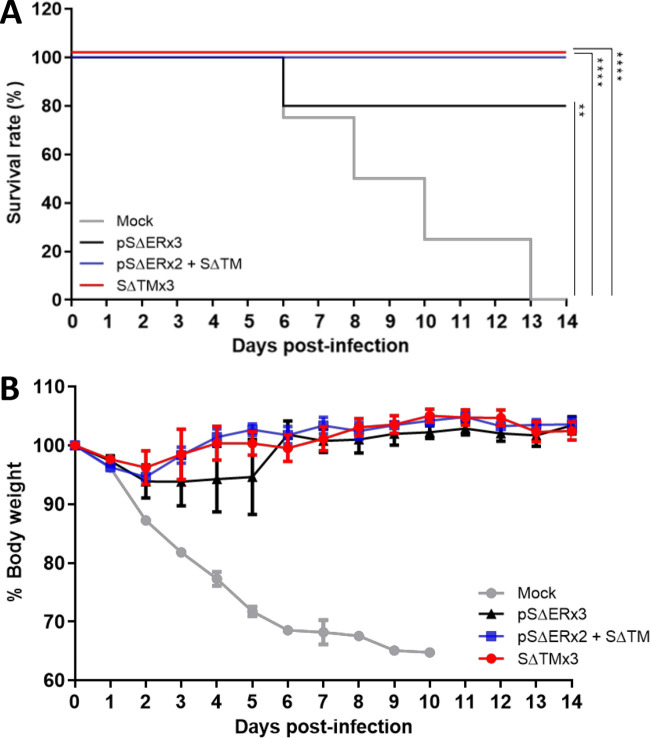
*In vivo* protective effect of MERS-CoV vaccine against wild-type MERS-CoV challenge. Immunization was performed using a total of 16 hDPP4 knock-in mice (7 to 9 weeks old). hDPP4 knock-in mice were i.m. immunized with pSΔER DNA two times, followed by SΔTM protein (*n *=* *4), pSΔER DNA (*n *=* *5), SΔTM protein (*n *=* *4), or PBS (*n *=* *3) as the negative control. All proteins were adjuvanted with alum hydroxide. Two weeks after the third immunization (at day 42), the mice were intranasally challenged with 2 × 10^5^ PFU of the wild-type MERS-CoV KOR/KNIH/002 strain. The survival rates (A) and body weight changes (B) for all mice were then monitored daily for 14 days.

MERS-CoV S-protein and human dipeptidyl peptidase 4 (DPP4) receptor-binding triggers the fusion of the virus and host cell membrane, and therefore the human DPP4 receptor is essential for MERS-CoV infection. However, the mouse DPP4 receptor does not play the same role as the human DPP4 receptor. To investigate the *in vivo* protective effect of our vaccine regimen against the wild-type MERS-CoV challenge, human DPP4 receptor knock-in mice were used. The mice were immunized with the pSΔER DNA prime-SΔTM protein boost vaccine, pSΔER DNA, or SΔTM protein three times biweekly. Two weeks after the last immunization, the vaccinated mice were challenged intranasally with 2 × 10^4^ PFU of the wild-type MERS-CoV KOR/KNIH/002 strain in the Animal Biological Safety Level 3 facility. Survival and body weight changes of each mouse were monitored daily for 14 days. As shown in Fig. 5A, none of the mice in the mock vector-immunized group survived against the wild-type MERS-CoV infection. On the other hand, all mice in the pSΔER DNA prime-SΔTM protein boost group and SΔTM protein-vaccinated group survived. A total of 80% (4/5) mice survived in the pSΔER DNA-vaccinated group (Fig. 5A). Significant body weight loss was observed in the mock vector-immunized mice. However, the mice that survived in the other vaccinated groups lost less than 10% of their initial body weight (Fig. 5B). These results demonstrated that pSΔER DNA prime-SΔTM protein boost vaccination provided protective immunity against wild-type MERS-CoV infection *in vivo*.

## DISCUSSION

In this study, we found that priming with DNA corresponding to *SΔER* and boosting with the recombinant SΔTM protein of the MERS-CoV EMC/2012 strain induces effective cross-neutralizing responses against 15 S-pseudovirions and cross-protection against the wild-type MERS-CoV KOR/KNIH/002 strain both *in vitro* and *in vivo*.

Coronavirus is known to have relatively higher genetic stability compared to other RNA viruses because of the proofreading function of nsp14 (15, 16). However, according to the analysis of the 2015 South Korean outbreak, the S gene, a target of vaccine development, was estimated to show a higher evolutionary rate with 6.72 × 10^−3^ substitutions/site/year (17) than that for complete MERV-CoV genomes with 1.12 × 10^−3^ substitutions per site per year or 9.29 × 10^−4^ substitutions/site/year (17, 18). Especially, aa 1020 of S, which is located in a domain required for cell entry, is known to be under strong positive selection (18). In addition, a few strains have been reported as possessing one or two mutations in the RBD of S (17, 18). Therefore, we speculated whether vaccine candidates derived from an S sequence of a single strain could provide cross-protection against different MERS-CoV S variants. To represent mutations in S, we constructed 12 RBD variants based on EMC/2012 S and 3 SΔER (EMC/2012, England1, and KOR/KNIH/002) pseudovirions and used them to examine the cross-neutralizing capability of vaccine candidates (14). In contrast to what we previously found, i.e., that several monoclonal antibodies cannot neutralize an S-pseudovirion containing even a single different amino acid in RBD (14), the polyclonal serum produced by our vaccine candidates (pSΔER DNA prime-SΔTM protein boost, pSΔER DNA, or SΔTM protein) exhibited cross-neutralization against all the 15 pseudovirions. The cross-protection activity of these vaccines against the wild-type KOR/KNIH/002 strain was also confirmed in PRNT and hDPP4 knock-in mice.

Antibody-dependent enhancement (ADE) of viral entry has been observed in a variety of viruses, such as dengue virus, HIV-1, Ebola virus, SARS-CoV-1, and MERS-CoV (6, 19, 20). The role of T cells remains less clear for exacerbated pathogenicity by ADE. However, some reports have suggested a protective role of CD8^+^ T cells in controlling Zika virus replication in mice and nonhuman primates (21, 22) and dengue virus replication in mice (23, 24). When we take T-cell-mediated immune responses by IFN-γ production into account, DNA priming-protein boosting vaccination is superior to protein immunization alone (25–27). While S1 and SΔTM protein immunization induced neutralizing antibodies against both pseudovirions and the wild-type MERS-CoV strain, they did not induce IFN-γ production in response to antigen peptide stimulation. Our results suggest that DNA priming-protein boosting is a promising strategy to induce both humoral and cellular immune responses than DNA or protein immunization alone. Previously, Al-Amri SS et al. reported immunogenicity of candidate MERS-CoV S vaccines; only mice immunized with pS and pS1 but not pSΔTM (1 to 1,295 aa) induced significant levels of S1-specific IgG compared to mock vector immunized group (8). Similarly, our pSΔTM (1 to 1,296 aa) induced a statistically significant lower level of RBD-specific IgG than pSΔER. It is not fully explained why pSΔER is superior to pSΔTM in inducing RBD-specific IgG in our study, but it might be due to a higher level of protein expression of SΔER and different level of major histocompatibility complex (MHC) class II presentation of their degraded proteins depending on protein processing characteristics in the ER or Golgi compartment (28).

Also, Wang L et al. reported full-length S DNA and S1 subunit protein expressed in mammalian cells as an approach for MERS-CoV vaccine development (13). However, our study used insect cell-expressed SΔTM subunit protein as the boosting antigen after DNA priming for MERS-CoV vaccine development. We did not compare the protective effect of S subunit proteins produced in mammalian cells and insect cells. However, insect cell-produced recombinant S1 and SΔTM efficiently boosted neutralizing immune responses in mice, although these proteins are known to exhibit different glycosylation patterns compared to the mammalian cell-expressed proteins. Interestingly, only RBD immunization induced a high level of binding antibodies but did not induce efficient neutralizing antibodies, suggesting that structural conformation is important for inducing neutralizing antibodies. Our results indicate that RBD conformation is critical for inducing neutralizing antibodies rather than glycosylated moieties in S, which is consistent with the observation that mammalian cell-expressed RBD is often fused to the human IgG Fc region or the foldon trimerization motif to function as a MERS-CoV vaccine candidate (29–31). Since SΔTM includes the S2 region that is involved in the fusion of the MERS-CoV virus to the host cell membrane, SΔTM may enhance the breadth of neutralizing antibodies than S1 (32). Therefore, we chose SΔTM for producing a booster vaccine and further investigated it: *SΔER* DNA priming-protein boosting with SΔTM provided a higher PRNT_50_ value against the wild-type MERS-CoV KOR/KNIH/002 strain than DNA immunization alone, which provided full protection against the wild-type KOR/KNIH/002 strain challenge in hDPP4 knock-in mice similar to SΔTM protein immunization alone. In summary, priming with pSΔER DNA and boosting with SΔTM protein is an effective way for MERS-CoV vaccine development by increasing both neutralizing antibody levels and cell-mediated immune responses. This study provides an important insight for selecting a suitable vaccine platform in developing vaccines against MERS-CoV or other emerging coronaviruses such as SARS-CoV-2.

## MATERIALS AND METHODS

### Animals.

Six-week-old female BALB/c (Koatech, Pyung Taek, South Korea) and C57BL/6 (Orient Bio, Sungnam, South Korea) mice were purchased and housed in the Animal Research Facility, International Vaccine Institute (IVI) under standard laboratory conditions. Human dipeptidyl protease 4 (hDPP4) knock-in mice were kindly provided by Paul McCray, University of Iowa, and bred at the IVI. Animal protocols were approved by the Institutional Animal Care and Use Committees of the IVI (2016-004 and 2017-004).

### Construction of MERS-CoV subunit S expression plasmids.

Human codon-optimized MERS-CoV S gene of the EMC/2012 isolate (GenBank accession number JX869059) (33) was used for preparing the PCR template for SΔER (deletion of the ER retention signal: 1 to 1,338 aa), SΔTM (1 to 1,296 aa), S1 (1 to 751 aa), S2 (752 to 1,296 aa), and RBD (358 to 606 aa) cloning with primer sets that contained a restriction enzyme site (SalI or BamHI). Details of the cloning primers are provided in Table 1. RBD and S2 included the S leader sequence of the EMC-2012 strain at the N terminus. Each PCR product was cloned into the mammalian expression vector pCMV/R 8κB (34) using SalI or BamHI sites. The gene encoding S of the KOR/KNIH/002 strain (GenBank accession number KT029139) was synthesized (GenScript, Piscataway, NJ). Other mutated genes encoding substituted residues in the RBD of S were generated by site-directed mutagenesis (QuikChange II XL site-directed mutagenesis kit; Agilent Technologies, Santa Clara, CA) using the EMC/2012 strain S gene as a template. The sequences of strains with mutations in MERS-CoV RBD residues were obtained from the GenBank database (14). All of the insert genes in the recombinant pCMV/R 8κB plasmids were verified by sequencing (Cosmo Genetech, Seoul, South Korea).

**TABLE 1:** Cloning primers for DNA vaccine constructs

Primer	Sequence (5′→3′)^*a*^
SΔER Forward	GTCGACATGATTCACTCTGTGTTCCTGC
SΔER Reverse	GGATCCTTAGTCGCAGCACCTGTTGC
SΔTM Forward	GTCGACATGATTCACTCTGTGTTCCTGC
SΔTM Reverse	GGATCCTTATGGCCACTTGTTGTAGTAG
S1 Forward	GTCGACATGATTCACTCTGTGTTCCTGC
S1 Reverse	GGATCCTTACCTCACAGACCTTGGTGTC
S2 Forward	GTCGACATGATTCACTCTGTGTTCCTGCTGATGTTCCTGCTGACACCAACAGAGTCCTATGTGTCTGTGCCTGGAGAGATG
S2 Reverse	GGATCCTTATGGCCACTTGTTGTAGTAG
RBD Forward	GTCGACATGATTCACTCTGTGTTCCTGCTGATGTTCCTGCTGACACCAACAGAGTCCTATGTGTCTGGAGTCTACTCTGTGTC
RBD Reverse	GGATCCTTAGTATTCCACACAGTTGCCAAG

a Restriction enzyme sites are underlined.

### Transfection and Western blot analysis.

HEK 293T cells (ATCC, CRL-3216) were transfected by using Lipofectamine 2000 transfection reagent (Thermo Fisher Scientific, Waltham, MA) according to the manufacturer’s recommendations, harvested 2 days after transfection, and maintained at −80°C. Cell lysates and culture supernatants were resolved by sodium dodecyl sulfate-polyacrylamide gel electrophoresis and transferred to a polyvinylidene fluoride membrane (Bio-Rad, Hercules, CA). The membrane was incubated with a 1:1,000-diluted sample of rabbit polyclonal IgG (Sino Biological, BDA, Beijing, China, catalog no. 40069-RP02) to detect SΔER, SΔTM, S1, S2, and RBD overnight at 4°C in blocking buffer (PBS [Thermo Fisher Scientific], 5% skim milk [BD Bioscience, San Jose, CA], 0.05% Tween 20 [Sigma, St. Louis, MO]), followed by washing. The blot was further incubated in a blocking buffer with 1:5,000 dilutions of horseradish peroxidase (HRP)-conjugated goat anti-rabbit IgG (Southern Biotech, Birmingham, AL) for 1 h at 22°C and then washed. Detection was performed with the enhanced chemiluminescence reagent (ELPIS-Biotech, Daejeon, South Korea).

### Mouse immunizations.

Six-week-old female BALB/c and C57BL/6 mice received MERS-CoV S plasmids (50 μg) three times at 2-week intervals or two times, followed by boosting with recombinant S subunit proteins (1 μg) adjuvanted with alum hydroxide (Thermo Fisher Scientific) i.m. Mouse sera were obtained after 14 days of each immunization under anesthesia (intraperitoneal route: ketamine hydrochloride [Yuhan Co., Ltd., Seoul, South Korea] and xylazine hydrochloride [Bayer Korea, Seoul, South Korea]).

### Enzyme-linked immunosorbent assay.

Recombinant MERS-CoV S subunit protein-specific antibody levels in the serum were measured by an enzyme-linked immunosorbent assay (ELISA). Ninety-six-well plates (Thermo Fisher Scientific) were coated with 100 ng/well of SΔTM, S1, or RBD recombinant protein in 100 μl of coating buffer (50 mM sodium bicarbonate [Sigma] in PBS [pH 9.6]) and incubated at 4°C overnight. After blocking with blocking buffer (1% bovine serum albumin [Merck, Darmstadt, Germany] in PBS) for 1 h at room temperature, serially diluted serum samples prepared in blocking buffer were added to the plates; they were then incubated for 1 h at 37°C. Next, HRP-conjugated goat anti-mouse IgG (1:3,000; Southern Biotech) was added, and the plates were incubated for 1 h at 37°C. After a final washing with wash buffer (0.05% Tween 20 in PBS), 3,3′,5,5′-tetramethylbenzidine liquid substrate solution (TMB; Millipore, Billerica, MA) was added (100 μl per well) for 1 to 2 min, and the reaction was then stopped by adding 1 N H_2_SO_4_ (Merck). The optical density was measured using an ELISA microplate reader (Molecular Devices, San Jose, CA). The antibody titer was expressed as the reciprocal log_2_ titer of dilution, showing an absorbance of 0.2 at 450 nm.

### Pseudovirus production.

HEK 293T/17 (ATCC, CRL-11268) cells were maintained in Dulbecco modified Eagle medium (DMEM; Thermo Fisher Scientific) supplemented with 10% fetal bovine serum (FBS; Thermo Fisher Scientific) and 1% penicillin/streptomycin (Thermo Fisher Scientific) and incubated at 37°C and 5% CO_2_. Cells at a concentration of 4 × 10^6^ were seeded into 100-mm dishes at a ratio that yielded 70 to 90% confluence at the time of transfection. Lentiviral pseudovirions expressing the MERS-CoV S-protein were produced by cotransfection of 293T cells with three plasmids, 7 μg of transducing plasmid pHR′CMV-Luc, 7 μg of packaging plasmid pCMV ΔR8.2 (35), and 100 ng of pCMV/R 8κB-SΔER plasmid of the MERS-CoV EMC/2012 or KOR/KNIH/002 strain, using Lipofectamine 2000 transfection reagent (Thermo Fisher Scientific). The medium was replaced after overnight incubation. Supernatants containing the pseudovirions were harvested 48 to 72 h after transfection, filtered through a 0.45-μm filter, and stored at −80°C. Titration of the pseudovirus was performed using a lentivirus-associated p24 ELISA kit (Cell Biolabs, San Diego, CA).

### Pseudovirus neutralization.

786-O cells (ATCC, CRL-1932) were plated at a density of 1 × 10^4^ cells/well in a 96-well plate with complete DMEM (10% FBS, 1% penicillin/streptomycin) at 37°C in a humidified atmosphere of 5% CO_2_ the day before pseudoviral infection until 95% confluence was reached. After incubation of 50 μl of 2 × 10^6^ pseudovirions with 20 μl of serially diluted mouse immune serum or neutralizing antibody as the positive control (Sino Biological) for 1 h at room temperature, the mixtures were added to the cells. The wells were replenished with 100 μl of fresh complete DMEM after 6 h of incubation. The cells were lysed after 72 h with 20 μl of lysis buffer (Promega, Madison, WI) and transferred to an opaque plate (Perkin-Elmer, Waltham, MA). Luciferase activity was measured by adding 40 μl of the substrate (Promega) and using a SpectraMax L microplate reader (Molecular Devices).

### IFN-γ ELISpot assay.

Immunized C57BL/6 mice were sacrificed at 14 days after they received the last immunization, and spleen lymphocytes were obtained from each mouse (36). Cells at concentrations of 1 × 10^6^, 3.3 × 10^5^, and 1.1 × 10^5^ were transferred to each well of precoated BD ELISpot plates (BD Bioscience). For stimulation, 5 μg/ml of MERS S pooled peptides (seven different MERS-CoV S peptides [S232, NH_2_-FNLRNCTFM-OH; S395, NH_2_-QVYNFKRL-OH; S434, NH_2_-ASNCYSSL-OH; S483, NH_2_-TVPHNLTTI-OH; S773, NH_2_-LNSSYFKL-OH; S945, NH_2_-AAYTSSLL-OH; and S1165, NH_2_-IAPVNGYFI-OH]) were added, followed by incubation for 16 h at 37°C in a 5% CO_2_ incubator. After discarding the cells and peptide stimulants and washing, biotinylated anti-mouse IFN-γ (BD Bioscience) was added to each well, and the plate was incubated at room temperature for 2 h. After the wells were washed, streptavidin-HRP (BD Bioscience) was added, and the plate was incubated at room temperature for 1 h. After washing, AEC substrate solution (BD AEC substrate reagent set; BD Biosciences) was added to each well. Spot development was stopped by washing the wells with deionized water, and spots were counted by using an ELISpot reader (Cellular Technology, Ltd., Cleveland, OH).

### Wild-type MERS-CoV neutralization.

VeroE6 cells were seeded at a density of 1 × 10^5^ cells/well in a 24-well plate with complete DMEM (10% FBS). The next day, the wild-type MERS-CoV KOR/KNIH/002 strain provided by Korea CDC was incubated with the serial dilutions of mouse sera at 37°C in a humidified atmosphere of 5% CO_2_ for 1 h. Then, the virus-serum mixtures were added to the cells, and 1 ml of overlay mixture (1% methyl cellulose; Sigma) in DMEM containing 10% FBS) was added after 1 h of incubation at 37°C. After 3 days, the cells were fixed with 4% paraformaldehyde (Intron Biotechnology, Dedham, MA) and permeabilized with methanol (Merck). The cells were incubated with rabbit anti-MERS-CoV N protein antibody (1:200; Sino Biological, Inc.) for 2 h at room temperature or overnight at 4°C, washed, and incubated with goat anti-rabbit alkaline phosphatase (1:10,000, Thermo Fisher Scientific) for 1 h at room temperature, followed by washing and incubation with the nitro-blue tetrazolium and 5-bromo-4-chloro-3′-indolyphosphate (NBT-BCIP) substrate solution (Merck) for 20 min. The plaques were counted, and the plaque reduction neutralization titer (PRNT_50_) was calculated.

### Wild-type MERS-CoV challenge.

hDPP4 knock-in mice were immunized two times with S (ΔER) DNA plasmid (50 μg) and boosted with alum adjuvanted S (ΔTM) recombinant protein (1 μg) at 2-week intervals. Control groups received the mock vector S (ΔER) DNA plasmid or S (ΔTM) protein three times. Two weeks after the last immunization, the mice were challenged with the MERS-CoV KOR/KNIH/002 strain (20,000 PFU; 50 μl) intranasally under anesthesia in the IVI BL3 lab. The survival and body weight of each mouse was monitored daily.

### Statistics.

All statistical analyses were performed using Prism 8 (GraphPad, San Diego, CA). Differences between individual groups were evaluated using one-way analysis of variance. Differences between the two groups were evaluated using a two-tailed Mann-Whitney U test. A log-rank (Mantel-Cox) test was used to compare the survival rates after the challenge. Two-tailed *P* values of <0.05 were considered statistically significant.
